# The efficacy and safety of CapeOX plus bevacizumab therapy followed by capecitabine plus bevacizumab maintenance therapy in patients with metastatic colorectal cancer: a multi-center, single-arm, phase II study (CCOG-0902)

**DOI:** 10.1186/s12885-017-3245-1

**Published:** 2017-04-04

**Authors:** Goro Nakayama, Kiyoshi Ishigure, Hiroyuki Yokoyama, Keisuke Uehara, Hiroshi Kojima, Akiharu Ishiyama, Naomi Hayashi, Nao Takano, Norifumi Hattori, Daisuke Kobayashi, Chie Tanaka, Masamichi Hayashi, Mitsuro Kanda, Suguru Yamada, Hiroyuki Sugimoto, Masahiko Koike, Michitaka Fujiwara, Tsutomu Fujii, Kenta Murotani, Yuichi Ando, Yasuhiro Kodera

**Affiliations:** 1 Department of Gastroenterological Surgery, Nagoya University Graduate School of Medicine, 65 Tsurumai-cho, Showa-ku, Nagoya, 466-8550 Japan; 2 Department of Surgery, Konan Kosei Hospital, Konan, Japan; 3 Department of Surgery, Komaki City Hospital, Komaki, Japan; 4 Department of Surgical Oncology, Nagoya University Graduate School of Medicine, Nagoya, Japan; 5 Department of Gastroenterological Surgery, Aichi Cancer Center Aichi Hospital, Okazaki, Japan; 6 Department of Surgery, Okazaki City Hospital, Okazaki, Japan; 7 Department of Surgery, Tosei Hospital, Seto, Japan; 8 Division of Biostatistics, Clinical Research Center, Aichi Medical University Hospital, Nagakute, Japan; 9 Department of Clinical Oncology and Chemotherapy, Nagoya University Hospital, Nagoya, Japan

**Keywords:** Metastatic colorectal cancer, Reintroduction of oxaliplatin, Maintenance therapy, Capecitabin, Bevacizumab, Peripheral sensory neuropathy

## Abstract

**Background:**

The aim of this study was to evaluate the efficacy and safety of CapeOX plus bevacizumab with a planned oxaliplatin stop-and-go strategy in Japanese patients with metastatic colorectal cancer (mCRC).

**Methods:**

Patients with untreated mCRC were treated with 4 cycles of CapeOX plus bevacizumab therapy, followed by capecitabine plus bevacizumab maintenance therapy. Reintroduction of oxaliplatin was scheduled after 8 cycles of maintenance therapy or upon tumor progression. The primary endpoint was progression-free survival (PFS), and secondary end points included overall survival (OS), objective response rate to each treatment, reintroduction rate of oxaliplatin, frequency of peripheral sensory neuropathy (PSN), and safety.

**Results:**

The 52 patients who received the protocol treatment were included in the evaluation of efficacy and safety. Median PFS and OS were 12.4 months (95% confidence interval [CI], 10.0–14.8) and 30.6 months (95% CI, 27.6–33.5), respectively. The objective response rates were 55.8% for the initial CapeOX plus bevacizumab therapy, 17.8% for capecitabine plus bevacizumab maintenance therapy, and 31.0% for reintroduced CapeOX plus bevacizumab therapy. The frequency of PSN was 63.5%, including 3.8% of patients with grade 3 PSN. No patients required treatment discontinuation because of PSN during the induction or maintenance therapy.

**Conclusions:**

CapeOX plus bevacizumab therapy with a planned oxaliplatin stop-and-go strategy is a feasible first-line treatment for Japanese patients with mCRC.

**Trial registration:**

This trial is registered with the University Hospital Medical Information Network in 15 March 2010 (UMIN000006478).

## Background

The first-line treatments for patients with metastatic colorectal cancer (mCRC) usually involves combination chemotherapies that include infusional 5-fluorouracil and leucovorin plus either irinotecan or oxaliplatin [1, 2]. Capecitabine, an oral fluoropyrimidine anticancer agent, in combination with oxaliplatin (CapeOX), had similar efficacy to regimens based on infusional 5-furuorouracil in combination with oxaliplatin used in previous studies [3–5]. The addition of bevacizumab, a humanized monoclonal antibody that inhibits vascular endothelial growth factor, to chemotherapy regimens that include CapeOX improves overall survival (OS) or progression-free survival (PFS) [5–7].

Peripheral sensory neuropathy (PSN), a cumulative dose-limiting toxicity of oxaliplatin, often requires the discontinuation of oxaliplatin before disease progression, and decreases patients’ quality of life. The results of several prospective studies have suggested the intermittent use of oxaliplatin to avoid its cumulative toxicity and prolong the time to treatment failure [8–11]. However, reports that describe details of the planned oxaliplatin stop-and-go strategy in bevacizumab containing regimens are limited [12–14].

The objectives of this study were (1) to evaluate the efficacy of CapeOX plus bevacizumab therapy with planned short-term initial CapeOX plus bevacizumab therapy, followed by 8 cycles of fixed-term maintenance therapy, and then the reintroduction of oxaliplatin for patients with mCRC, and (2) to assess the safety, including the incidence of PSN, of this strategy.

## Methods

### Study design and patients eligibility

This multicenter, single-arm, phase 2 trial was conducted by the Chubu Clinical Oncology Group (CCOG) in 18 hospitals of Japan. The criteria for inclusion in this study were age at least 20 years; histologically proven adenocarcinoma of the colon or rectum; unresectable metastasis; no previous chemotherapy for metastatic disease; at least one measurable lesion according to the Response Evaluation Criteria in Solid Tumors (RECIST), version 1.1; an Eastern Cooperative Oncology Group (ECOG) Performance Status of 0 or 1; and adequate bone marrow, liver, and renal functions. Patients may register if they have received adjuvant chemotherapy with fluoropyrimidine and cancer recurrence has occurred 6 months or more after the last dose. Patients who have received oxaliplatin-based adjuvant chemotherapy may not register. Patients with brain metastasis, clinically significant cardiovascular disease, second malignancies, bowel obstruction, PNS more than grade 1, uncontrolled diabetes mellitus or hypertension were excluded. Patients completed written informed consent before participating, and the ethics committees of Nagoya University Hospital and each participating facility approved the study. This trial was registered with the University Hospital Medical Information Network in 15 March 2010 (UMIN000006478).

### Treatment plan

Induction therapy: Patients received CapeOX plus bevacizumab therapy for 4 cycles, which consisted of intravenous oxaliplatin (130 mg/m^2^) and bevacizumab (7.5 mg/kg) on day 1 in combination with oral capecitabine (1000 mg/m^2^ twice daily) given as intermittent treatment for 14 days followed by 7 days treatment-free interval, every 3 weeks. Maintenance therapy: Capecitabine plus bevacizumab therapy which consisted of intravenous administration of bevacizumab (7.5 mg/kg) on day 1 and capecitabine (1000 mg/m^2^ twice daily), was initiated for patients with stable disease or a superior response after 4 cycles of the induction therapy. Reintroduction therapy: Reintroduction of oxaliplatin was scheduled after 8 cycles of the maintenance therapy. Oxaliplatin was also reintroduced in the event of tumor progression before 8 cycles of the maintenance therapy (in a case where tumor progression was observed before 8 cycles of the maintenance therapy). The reintroduction therapy continued until disease progression, unacceptable toxicity, or the patient refusal.

### Measurements

The primary objective of this study was PFS, defined as the time from the date therapy was initiated until the date of disease progression or death from any cause. The secondary objectives were the following: OS, defined as the time from the date that therapy was initiated until the date of death from to any cause; duration of disease control (DDC), defined as PFS in the patients without disease progression before oxaliplatin reintroduction or the patients with progression at the first evaluation after oxaliplatin reintroduction. In the case achieved disease progression before oxaliplatin reintroduction and tumor response or stabilization at the first evaluation after oxaliplatin reintroduction, DDC was defined as the sum of the initial PFS and the PFS of the reintroduction [8]; overall response rate (ORR), defined as the proportion of patients whose best response was complete response (CR) or partial response (PR); disease control rate (DCR), defined as the proportion of patients whose best response was CR, PR or stable disease (SD); reintroduction rate of oxaliplatin, defined as the proportion of patients who received the reintroduced CapeOX plus bevacizumab therapy; and the incidence of adverse events, including the frequency and severity of PSN. Tumor size and response according to RECIST, version 1.1, based on chest-to-pelvic region computed tomography (CT) once every 8 weeks were evaluated by the local review in each participating facility. Adverse events were assessed using National Cancer Institute Common Toxicity Criteria (NCI-CTC), version 3.0.

### Statistical analysis

A power analysis was conducted before the study. Assuming that the threshold for PFS was 7.2 months and the expected PFS was 10.4 months, referring to data from the previous clinical trials [4, 7], with the enrollment period of 2 years and the follow-up period of 3 years, 47 patients were required to ensure an alpha level of 0.05 (one-sided) and a detection power (1-β) of 80%. The sample size for this study was 50 to account for possible loss to follow-up.

The PFS, the primary objective of this study, was estimated using the Kaplan–Meier method, and the median PFS and its 95% confidence interval were estimated. Other time-to-endpoint variables, DDC and OS, were estimated using the same method. The ORR, DCR and incidence the toxicities were calculated as proportions with exact confidence intervals. Statistical analyses were performed using SPSS, version 23 (SPSS Inc., Chicago, IL, USA).

## Results

### Patient characteristics

Fifty-four patients from 18 institutions were enrolled in this study between April 2010 and October 2011. Two patients were excluded after enrollment because of ineligibility. The remaining 52 patients who received the protocol treatment were included in the evaluation of efficacy and safety. Baseline characteristics of the 52 patients are presented in Table 1.

**Table 1:** Characteristics of patients

Variable	*N* = 52	
	n	%
Sex		
Male	31	59.6
Female	21	40.4
Age, years		
Median (range)	66 (40–80)	
Performance status WHO		
0	38	73.1
1	14	26.9
Primary site		
Colon	28	53.8
Rectum	24	46.2
Metastases		
Synchronous	18	34.6
Metachronous	34	65.4
Nunmer of metastatic site		
1	37	71.2
>1	15	28.8
Metastaic sites		
Liver	30	57.7
Liver only	16	30.8
Lung	20	38.5
Peritoneum	9	9.6
Lymph nodes	5	17.3
Prior treatment		
Adjuvant chemotherapy^a^	20	38.5
Surgery^b^	35	67.3
Radiotheray	0	0
*KRAS* status		
Wild type	24/43	55.8
Mutant type	19/43	44.2

*N* total number of patients, *n* number of patients, *WHO* World Health Organization

^a^Chemotherapy with fluoropyrimidine. No patients received oxaliplatin-based adjuvant chemotherapy

^b^Resection of primary site

### Treatment status

**Fig. 1:**
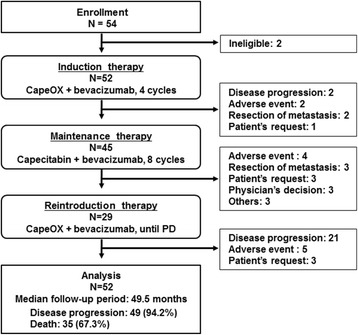
Consort chart of study participants. Fifty-two patients enrolled between April 2010 and October 2011 received the initial CapeOX plus bevacizumab therapy. The maintenance therapy with capecitabin and bevacizumab was introduced in 45 patients, and oxaliplatine was reintroduced in 29 patients. N, number of patients; CapeOX, capecitabin and oxaliplatin; PD, progression disease

The initial CapeOX plus bevacizumab therapy was administered to 52 patients, including 50 patients who accomplish 4 cycles of the induction therapy. The maintenance therapy, capecitabin and bevacizumab, was administered to 45 patients (86.5%). Twenty-two of those patients could accomplish 8 cycles of maintenance therapy. Oxaliplatine was reintroduced in 29 patients (55.8%), including 20 patients accomplished 8 cycles of the maintenance therapy and 9 patients with disease progression during the maintenance therapy. A consort chart of patients is presented in Fig. 1. The median number of treatment cycles was 4 (1–4 cycles) for the initial CapeOX plus bevacizumab therapy, 7 (1–8 cycles) for the capecitabin plus bevacizumab maintenance therapy, and 5 (1–22 cycles) for the reintroduced CapeOX plus bevacizumab therapy. The median time-to-treatment failure of the protocol treatment was 9.9 months (95% confidence interval [CI]: 5.9–13.8 months).

The relative dose intensity of oxaliplatin in the initial and reintroduced CapeOX plus bevacizumab therapy were 92.3 and 78.5%, respectively, and the cumulative dose of oxaliplatin during the overall treatment period was 1052 mg (470–4346 mg). The protocol treatment was discontinued because of the following circumstances: disease progression in 28 patients (53.8%), adverse events in 11 (21.2%), and resection of metastasis in 5 (9.6%).

Treatment status is summarized in Table 2.

**Table 2:** Treatment status of patients

	Induction therapy CapeOX + BEV (*N* = 52)	Maintenance therapy Capecitabin + BEV (*N* = 45)	Reintoroduction therapy CapeOX + BEV (*N* = 29)
Treatment cycle, times			
Median (range)	4 (2–4)	7 (1–8)	5 (1–21)
Median relative dose-intensity, %			
Oxaliplatin	92.3	-	78.5
Capecitabin	92.0	82.4	71.8
Bevacizumab	96.6	92.0	91.0
Total dose of oxaliplatin, mg			
Median (range)	1052 (470–4346)		
Time-to-treatment failuer, months			
Median (95% CI)	9.9 (5.9–13.8)		
Post progression treatment, n (%)			
Second-line therapy	46	(88.5)	
Anti-EGFR agents	17	(32.7)	
Bevacizumab (BBP)	35	(67.3)	
Resection of metastasis	8	(15.4)	

*BEV* bevacizumab, *N* total number of patients, *CI* confidence interval, *BBP* Bevacizumab beyond progression

### Treatment outcomes

**Fig. 2:**
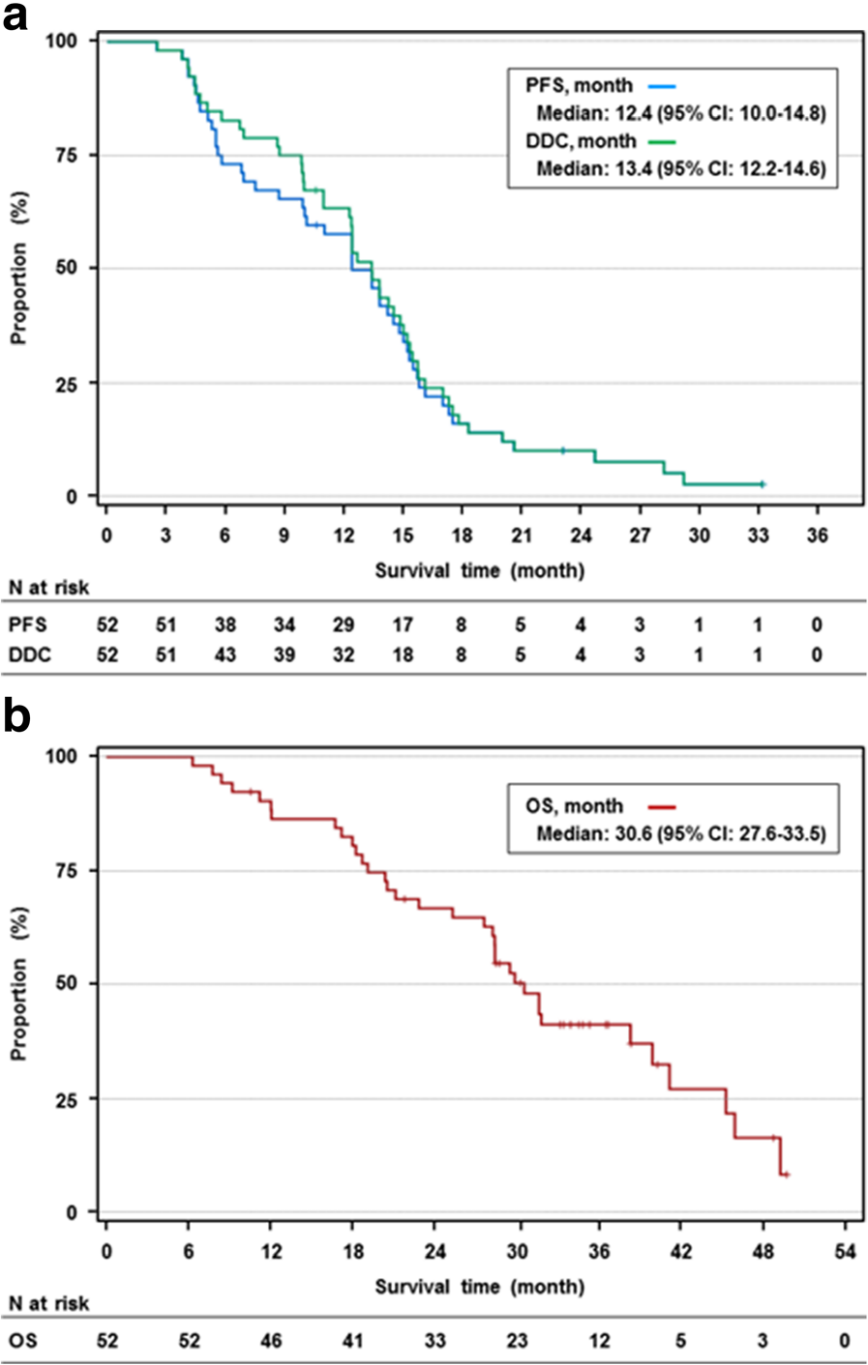
**a.** Kaplan–Meier analysis of progression-free survival and duration of disease control. Median progression-free survival and duration of disease control were 12.4 months (95% CI: 10.0–14.8 months) and 13.4 months (95% CI: 12.2–14.6 months), respectively. PFS, progression-free survival; DDC, duration of disease control; CI, confidence interval; N, number of patients. **b.** Kaplan–Meier analysis of overall survival. Median overall survival was 30.6 months (95% CI: 27.6–33.5 months). OS, overall survival, OS; CI, confidence interval; N, number of patients

After a median follow-up period of 49.5 months (range: 6.3–63.9 months), the disease progressed in 49 patients (94.2%) and 35 deaths (67.3%) occurred in the 52 patients enrolled. Median PFS, the primary endpoint, and DDC were 12.4 months (95% CI, 10.0–14.8 months) and 13.4 months (95% CI, 12.2–14.6 months), respectively (Fig. 2a). There was no significant difference between these two survival outcomes (HR 0.932, 95% CI 0.627–1.385, *p* = 0.727). The median OS was 30.6 months (95% CI, 27.6–33.5 months) (Fig. 2b). The ORR for the initial CapeOX plus bevacizumab therapy, maintenance capecitabin plus bevacizumab therapy, and reintroduced CapeOX plus bevacizumab therapy were 55.8, 17.8, and 31.0%, respectively. The DCR for those therapies were 96.2, 80.0, and 89.7%, respectively. The tumor response to each phase of treatment is summarized in Table 3.

**Table 3:** Objective tumor response

	Induction therapy CapeOX + BEV (*N* = 52)		Maintenance therapy Capecitabin + BEV (*N* = 45)		Reintoroduction therapy CapeOX + BEV (*N* = 29)	
	n	%	n	%	n	%
CR	0	0	0	0	2	6.9
PR	29	55.8	8	17.8	7	24.1
SD	21	40.4	28	62.2	17	58.6
PD	2	3.8	9	20.0	3	10.3
ORR, %	55.8		17.8		31.0	
DCR, %	96.2		80.0		89.7	

*BEV* bevacizumab, *N* total number of patients, *n* number of patients, *CR* complete response, *PR* partial response, *SD* stable disease, *PD* progressive disease, *ORR* objective response rate ([CR + PR]/N × 100), *DCR* disease control rate ([CR + PR + SD]/N × 100)

### Adverse events

**Fig. 3:**
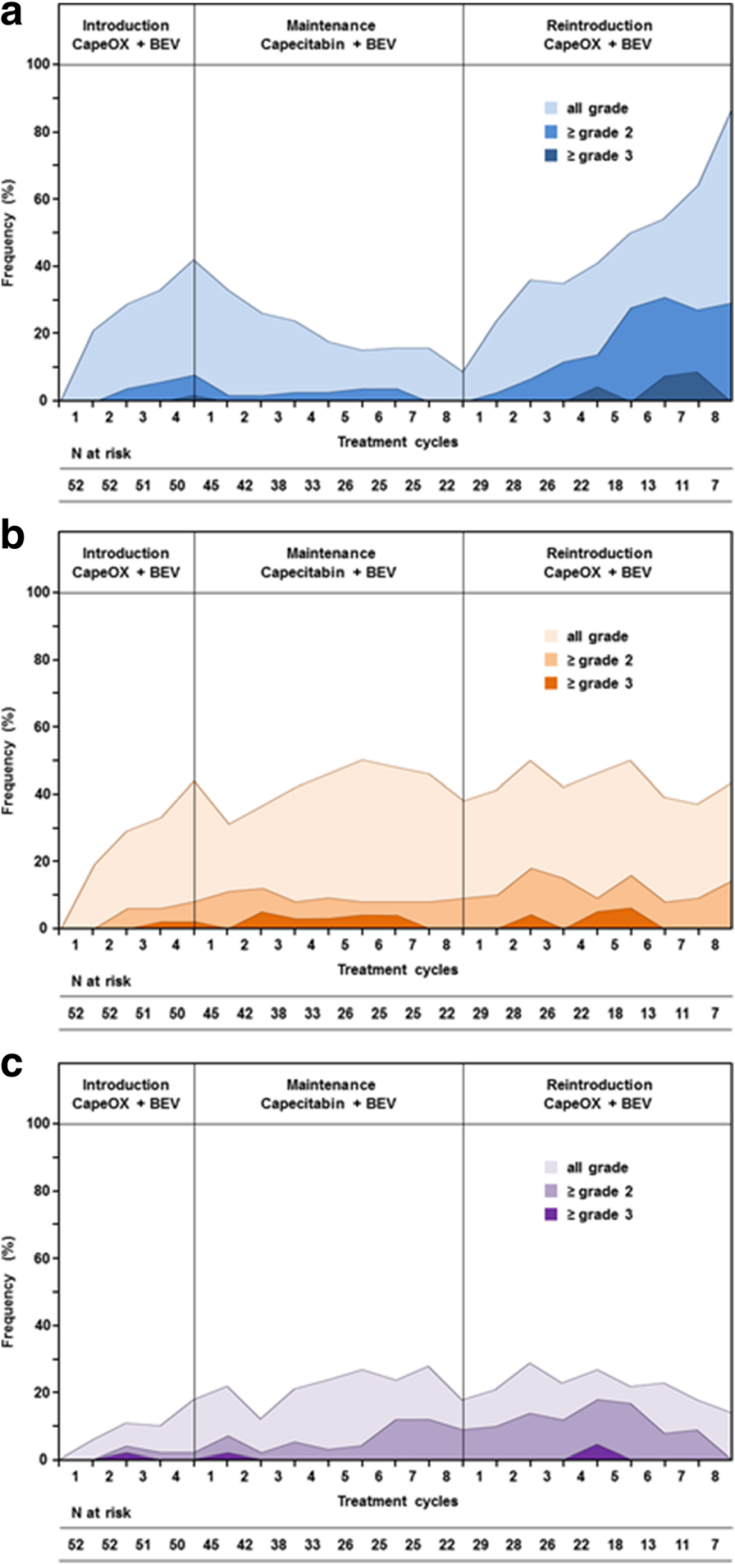
**a**. Incidence of peripheral sensory neuropathy. The cumulative peripheral sensory neuropathy rate was 63.5%, including 3.8% of patients with grade 3. The frequencies of PNS after 4 cycles of initial CapeOX plus bevacizumab therapy and 8 cycles of the maintenance therapy with capecitabin plus bevacizumab were 42 and 9%, respectively. CapeOX, capecitabin and oxaliplatin; BEV, bevacizumab; N, total number of patients. **b**. Incidence of hand-foot syndrome. The frequencies of hand-foot syndrome were 63.5% in induction therapy, 48.9% in maintenance therapy, and 55.2% in reintroduced therapy. CapeOX, capecitabin and oxaliplatin; BEV, bevacizumab; N, total number of patients. **c**. Incidence of bevacizumab-related toxicities. The frequencies of bevacizumab-related toxicities were 26.9% in induction therapy, 37.8% in maintenance therapy, and 37.9% in reintroduced therapy. CapeOX, capecitabin and oxaliplatin; BEV, bevacizumab; N, total number of patients

The incidence of treatment-related adverse events is presented in Table 4. The frequencies of hematological and non-hematological adverse events above grade three were 13.5 and 38.5%, respectively, for the overall treatment period. PSN occurred in 33 patients (63.5%), including two patients (3.8%) with PSN grade 3. Grade-3 PSN occurred in one patient at the end of the induction therapy and one patient after fourth cycle of the reintroduction therapy. The frequencies of PNS after 4 cycles of the initial CapeOX plus bevacizumab therapy and after 8 cycles of the maintenance therapy were 42 and 9%, respectively (Fig. 3). During the induction and maintenance therapy, no patients required treatment discontinuation due to PSN. The incidence of hand-foot syndrome and bevacizumab-related toxicities is presented in Fig. 3a and b, respectively. These toxicities occurred in relatively constant rate of patients throughout all treatment period, however, treatment was discontinued in 3 patients (5.8%) due to HFS and 4 patients (7.7%) due to bevacizumab-related toxicities.

**Table 4:** Occurrence of common toxicities

Toxicity	Induction therapy CapeOX + BEV (*N* = 52)				Maintenance therapy Capecitabin + BEV (*N* = 45)				Reintoroduction therapy CapeOX + BEV (*N* = 29)			
	All grade		≥Grade 3		All grade		≥Grade 3		All grade		≥Grade 3	
	n	%	n	%	n	%	n	%	n	%	n	%
Hematologic toxiciy	20	38.5	5	9.6	12	26.7	3	6.7	11	37.9	2	6.9
Neutropenia	10	19.2	4	7.7	4	8.9	2	4.4	4	13.8	2	6.9
Thrombocytopenia	9	17.3	0	0	8	17.8	1	2.2	8	27.6	0	0
Anemia	9	17.3	0	0	4	8.9	0	0	3	7.1	0	0
Febrile neutropenia	1	1.9	1	1.9	0	0	0	0	0	0	0	0
Non-hematologic toxicity	44	84.6	8	15.4	36	80.0	6	13.3	22	75.9	6	20.7
Diarrhea	7	13.5	1	1.9	2	4.4	0	0	2	6.9	0	0
Nausea/vomiting	8	15.4	0	0	2	4.4	0	0	3	10.3	0	0
Mucositis	8	15.4	0	0	2	4.4	0	0	2	6.9	0	0
Hand-foot syndrome	33	63.5	1	1.9	22	48.9	4	8.9	16	55.2	3	10.3
Fatigue	0	0	0	0	1	2.2	0	0	1	3.4	0	0
Peripheral neuropathy	26	50.0	1	1.9	17	37.8	0	0	14	48.3	2	6.9
Allergy	2	3.8	1	1.9	0	0	0	0	3	10.3	2	6.9
BEV-related toxicities	14	26.9	1	1.9	17	37.8	1	2.2	11	37.9	1	3.4
Hypertension	9	17.3	0	0	11	24.4	0	0	8	27.6	0	0
Proteinuria	6	11.5	0	0	5	11.1	0	0	3	10.3	1	3.4
Bleeding	0	0	0	0	0	0	0	0	1	3.4	0	0
Infection	1	1.9	0	0	2	4.4	1	2.2	1	3.4	0	0
Thrombosis	1	1.9	1	1.9	0	0	0	0	0	0	0	0

*BEV* bevacizumab, *N* total number of patients, *n* number of patients

## Discussion

The present phase II study was conducted to evaluate the efficacy and safety of CapeOX plus bevacizumab with a planned stop-and-go strategy as the first-line setting in Japanese patients with mCRC. The median PFS, the primary endpoint, was 12.4 months, which was considered acceptable according to the formulation of our hypothesis that assumes the threshold and expected survival time of 7.2 months and 10.4 months, respectively. Furthermore, the other survival outcomes including OS of 30.6 months and DCC of 13.4 months were comparable to those reported in several studies evaluating oxaliplatin-based chemotherapies plus bevacizumab with intermittent use of oxaliplatin [12–14].

Fifty percent of our patients experienced PSN during initial CapeOX plus bevacizumab therapy, with a median cumulative oxaliplatin dose of 782 mg. However, after 8 cycles of maintenance therapy, the frequency of PSN decreased to 9.1%. No patients required treatment discontinuation due to PSN during the initial CapeOX plus bevacizumab therapy or the maintenance therapy. In addition, severe PSN of grade 3 or higher was observed in only 3.8% of patients throughout the treatment period. These results were comparable to those from previous studies [8, 12–14], and might have contributed to the longer time-to-treatment failure of 9.9 months in our current study.

The design of our treatment protocol included a brief induction therapy of 4 cycles with CapeOX plus bevacizumab followed by fixed-term maintenance therapy of 8 cycles with capecitabin plus bevacizumab. This differed from those in previously reported studies, which mostly included 6 cycles of induction therapy followed by maintenance therapy until disease progression [9, 12–14]. In the CAIRO3 trial, a large randomized trial evaluating maintenance treatment with capecitabin plus bevacizumab, the reintroduction rate of oxaliplatin was 47% in patients receiving the maintenance therapy [14]. The overall reintroduction rates were reported 40–50% in several other studies testing intermittent use of oxaliplatin [8, 9, 11–13]. In our study, oxaliplatin was reintroduced in 55.8% of the whole cohort, and 64.4% of patients who received maintenance therapy could tolerate reintroduction, achieving a 28% objective response and 83% disease control during the reintroduction phase. In addition, the 22 patients (43%) who could accomplish 8 cycles of maintenance therapy had achieved relatively good prognosis, with 15.0 months of PFS and 38.3 months of OS. Although this study was not randomized and the consideration of several biases due to disease biology should be required, these results suggest that our treatment plan could be feasible as an intermitted oxaliplatin treatment strategy.

In the E3200 trial, which investigated the addition of bevacizumab to FOLFOX in 829 patients with previously treated mCRC, the incidence of PSN with grade 3 or higher was significantly higher in the bevacizumab arm, and authors attributed the exacerbation of oxaliplatin-induced PSN to a longer duration of chemotherapy or higher cumulative dose in the bevacizumab arm [15]. In our previous study, CCOG-0704 evaluating FOLFOX with an oral fluoropyrimidine maintenance therapy, the median PFS and OS were 7.4 and 28.0 months, respectively, and the incidence of PSN was much higher at 93.3% [11], although the dose intensity of oxaliplatin was similar to that in the present study. Although the designs of our studies do not allow for a quantified comparison, these results suggest that the addition of bevacizumab to cytotoxic regimens may contribute to survival benefits, and at least may not exacerbate oxaliplatin-induced PSN.

This study has several limitations. First, tumour size and response according to RECIST were not evaluated by central review. Second, the one-arm design and relatively small sample size of this study necessitates confirmation of these results in a larger cohort study. However, it does imply that this strategy, with a brief induction therapy with CapeOX plus bevacizumab, followed by a fixed-term maintenance therapy is feasible for the Japanese patients with mCRC. Data regarding the safety of this strategy was more robust, especially the incidence of PNS. From these encouraging data, it can now be recommended that a randomized controlled trial involving a larger numbers of patients be performed in Japan to obtain more robust and detailed data regarding the efficacy of this strategy and the continued use of bevacizumab.

## Conclusion

In summary, the planned oxaliplatin stop-and-go strategy with a brief induction therapy of CapeOX plus bevacizumab and maintenance therapy of capecitabin plus bevacizumab is feasible for the Japanese mCRC patients.

## Abbreviations

mCRC: Metastatic colorectal cancer
CapeOX: Capecitabine and oxaliplatin
OS: Overall survival
PFS: Progression-free survival
PNS: Peripheral sensory neuropathy
CCOG: Chubu Clinical Oncology Group
RECIST: Response Evaluation Criteria in Solid Tumors
ECOG: Eastern Cooperative Oncology Group
UMIN: University Hospital Medical Information Network
DDC: Duration of disease control
ORR: Overall response rate
CR: Complete response
PR: Partial response
DCR: Disease control rate
SD: Stable disease
NCI-CTC: National Cancer Institute Common Toxicity Criteria
CI: Confidence interval
